# Competition of single and double rescattering in the strong-field photoemission from dielectric nanospheres

**DOI:** 10.1007/s00340-016-6369-0

**Published:** 2016-04-12

**Authors:** L. Seiffert, F. Süßmann, S. Zherebtsov, P. Rupp, C. Peltz, E. Rühl, M. F. Kling, T. Fennel

**Affiliations:** 1 Institut für Physik, Universität Rostock, 18051 Rostock, Germany; 2 Max-Planck-Institut für Quantenoptik, 85748 Garching, Germany; 3 Physik Department, Ludwig-Maximilians-Universität München, 85748 Garching, Germany; 4 Physical Chemistry, Freie Universität Berlin, 14195 Berlin, Germany

**Keywords:** Trapping Potential, Large Sphere, Tangential Field, Sphere Size, Tunnel Ionization

## Abstract

Nanostructures exposed to ultrashort waveform-controlled laser pulses enable the generation of enhanced and highly localized near fields with adjustable local electric field evolution. Here, we study dielectric SiO_2_ nanospheres (*d* = 100–700 nm) under strong carrier-envelope phase-controlled few-cycle laser pulses and perform a systematic theoretical analysis of the resulting near-field driven photoemission. In particular, we analyze the impacts of charge interaction and local field ellipticity on the near-field driven electron acceleration. Our semiclassical transport simulations predict strong quenching of the electron emission and enhanced electron energies due to the ionization induced space charge. Though single surface backscattering remains the main emission process for the considered parameter range, we find a substantial contribution of double rescattering that increases with sphere size and becomes dominant near the cutoff energy for the largest investigated spheres. The growing importance of the double recollision process is traced back to the increasing local field ellipticity via trajectory analysis and the corresponding initial to final state correlation. Finally, we compare the carrier-envelope phase-dependent emission of single and double recollision electrons and find that both exhibit a characteristic directional switching behavior.

## Introduction

The excitation of nanostructures with laser light can be utilized to generate near fields that are localized on sub-wavelength scales and exhibit strong-field enhancement [1]. Controlling the structure of near fields with nanometer spatial and attosecond temporal resolution promises precise steering of the strong-field induced electron motion and is therefore of key interest for the realization of ultrafast light-driven nanoelectronics [2]. The feasibility of using nonresonant near fields for the control of strong-field electron dynamics with high time resolution has been demonstrated in photoemission experiments on metal nanotips [3–7], isolated dielectric nanospheres [8–10], and surface-assembled nanoantennas [11]. It has been shown that, similar to atomic above threshold ionization [12], electron backscattering of tunnel electrons from the nanostructure’s surface dominates the high-energy electron emission and can be controlled by the carrier-envelope phase (CEP) of the incident light field [3, 5, 8–10]. The importance of the spatial field profile has been shown by the observed quenching of backscattering if the quiver amplitude exceeds the near-field extension [4]. In this impulsive acceleration regime, also spectral focussing and defocussing via an additional THz field has been demonstrated in the strong-field emission from biased nanotips [13]. Near fields have also been used to drive quantum-coherent electronic free–free transitions of high-energy electrons [14], in analogy to two-color atomic strong-field ionization of atoms [15].

**Fig. 1:**
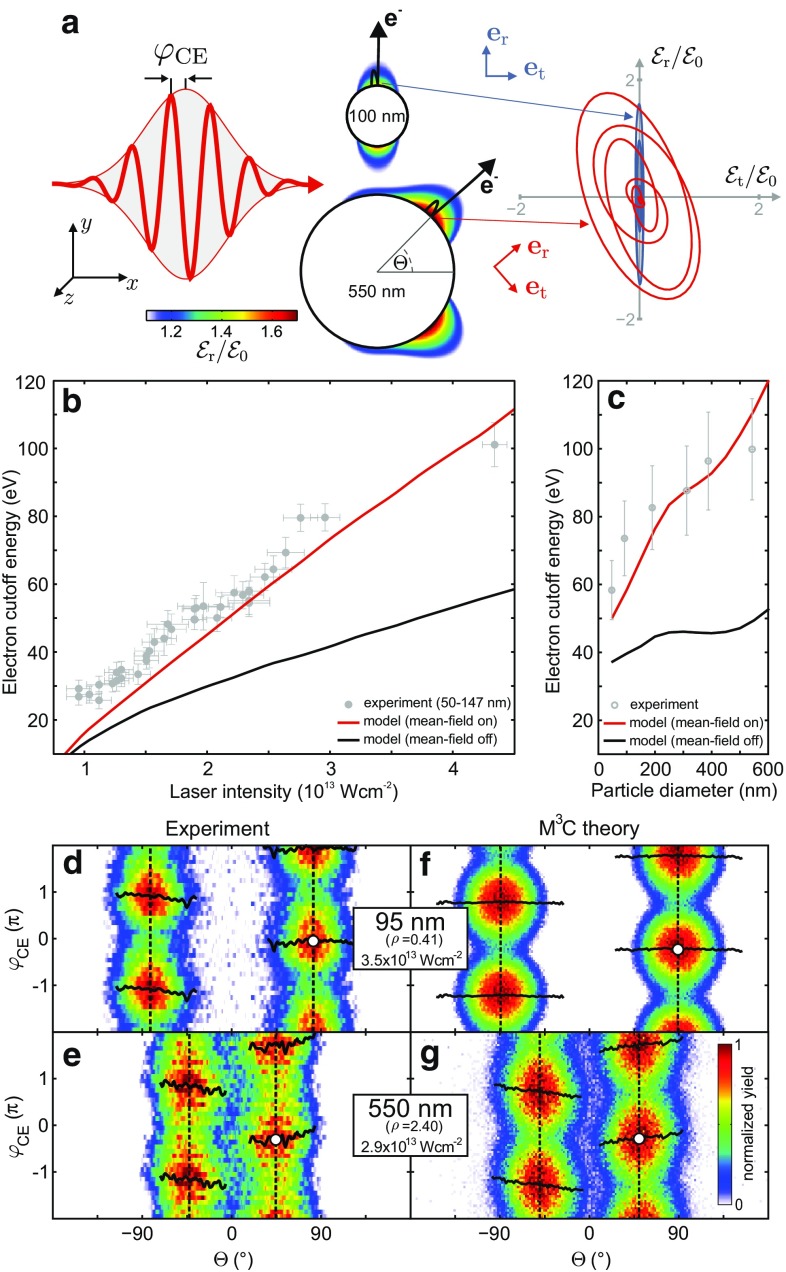
Electron emission from SiO_2_ spheres. **a** Mie solution for the peak radial near-field enhancement of small and large spheres calculated for 4 fs linearly polarized few-cycle laser pulses at 720 nm and $\varphi _\text {CE} = 0$ that propagate in *x*-direction. Enhancement profiles are shown for the *x*–*y* plane at $z=0$. The vectorial field evolution (*right*) corresponds to the local reference frame in the respective hot spots (see radial and tangential unit vectors). **b** Measured laser intensity dependence of the electron cutoff energy from small ($d\approx 100 \pm 50\,\hbox {nm}$) spheres (*gray symbols*) and corresponding simulation results without (*black curve*) and with mean field (*red curve*) for $d = 100\,\hbox {nm}$ particles. Note that these results are adapted from [8] and have been obtained for $5\,\hbox {fs}$ pulses. **c** Cutoff energy evolution with sphere size at $I=3.0 \times 10^{13}\,\hbox {Wcm}^{-2}$ for $4\,\hbox {fs}$ pulses . *Gray dots* represent the measurements while *black* and *red curves* show simulation results neglecting and including the mean field, respectively. Adapted from [10]. Slight discrepancies between the cutoffs in **b** and **c** are mainly attributed to the different pulse length. **d**–**g** Directionality and phase-dependent switching of measured and calculated yields from fast electrons with $E>E_\text{th} = 0.5E_\text{c}$, where $E_\text {c}$ is the cutoff energy. The definition of the cutoff energy in the simulations is described in Fig. 2. In the experiments, the cutoff corresponds to the energy up to which CEP-dependent signal is observed. Sphere sizes and laser intensities as indicated. Critical upward and downward emission angles $\varTheta^\text {crit}$ (*vertical dashed lines*) characterize the maxima of the amplitude $A(\theta )$ from harmonic fits of the yields with $Y(\theta ,\varphi _\text {CE}) = Y_0(\theta ) + A(\theta )\cos (\varphi _\text {CE} - \Delta \varphi (\theta ))$. *White dots* indicate the critical CEP $\varphi _\text {CE}^\text {crit} = \Delta \varphi (\varTheta ^\text {crit})$ for maximal upward emission under the critical angle. Adapted from [10]

Two important effects that have distinct impacts on the near-field driven electron dynamics are (1) the feedback from charge interaction for multielectron emission and (2) electromagnetic field propagation. Preceding studies have shown that the measured cutoff energies of recollision electrons emitted from small dielectric SiO_2_ spheres ($d\approx 100\,\hbox {nm}$) increase linearly with the intensity of a driving infrared few-cycle pulse [8], as shown in Fig. 1b. Note that for such small spheres the field propagation can still be neglected and the hot spots occur at the particle poles (Fig. 1a). However, the observed energies are about a factor of two higher than the prediction of classical rescattering in the linear near field alone. Quasi-classical trajectory Monte Carlo simulations could explain the enhanced cutoff by the effect of charge interaction, which supports the acceleration process via the emerging local trapping potential at the particle surface and Coulomb repulsion in the escaping electron bunch (compare black and red curves in Fig. 1b).

More recently, the effect of field propagation on the emission dynamics has been studied by varying the sphere size [10]. While the cutoff energy enhancement induced by charge interaction increases even further with size (Fig. 1c), the field propagation induced spatial deformation of the near field for larger spheres is directly mapped into the directionality of the electron emission, as shown in Fig. 1d,e. The comparison of the angular-resolved yield of fast electrons for small and large spheres shows a tilt of the main emission direction by about 45 degrees toward the backside for large particles. The excellent agreement with trajectory-based transport simulations including the field propagation effect (see Fig. 1f,g) supports the prevalence of single backscattering and a strong CEP dependence that enables controlled switching of the emission yield into the upper or lower half-space. It should be noted that the linear near field (Mie solution) in the hot spot region of large spheres exhibits substantial tangential components [10], resulting in a pronounced local field ellipticity (Fig. 1a). For single rescattering electrons, which dominate the high-energy emission in the so far investigated particle size range ($\lesssim 500\,\hbox {nm}$), however, the ellipticity effect was found to be small [10]. Here, we report simulation results for even larger particles, where we find a so far unreported qualitative change in the electron acceleration process. In particular, we find that the further increased near-field ellipticity leads to a particularly strong enhancement of double rescattering, which eventually becomes dominant for the high-energy photoemission.

The major objective of the current study is to unravel the mechanism underlying the enhancement of the double recollision process. Further, we systematically analyze the intensity and sphere size dependence of the near-field driven photoemission to address the effects of charge interaction and field ellipticity, respectively. We consider SiO_2_ spheres with diameters in the range of $d = 100-700\,\hbox {nm}$ under intense CEP-controlled few-cycle laser pulses. Our theoretical analysis based on quasi-classical simulations reveals the following main results. We find that the yield and cutoff energy of recollision electrons scale almost linearly with laser intensity for all investigated sphere sizes. The scaling of the yield with sphere size can be explained with Coulomb trapping by the attractive space charge potential of the ionized spheres. Furthermore, we find that the double recollision process becomes increasingly important for large spheres and begins to dominate the electron spectra near the cutoff for $d\gtrsim 600\,\hbox {nm}$ for the considered laser parameters. Via trajectory analysis, we show that the field ellipticity is responsible for the increasing significance of double recollision electrons. Finally, the implications of the competition of single and double recollision electrons for the CEP-dependent emission are analyzed. While the phase-dependent switching behavior of the two processes is qualitatively similar, we find characteristic differences in the angular and energy dependence that provide a basis for an experimental discrimination of the two photoemission channels.

## Theoretical framework

The electron dynamics at the dielectric nanospheres are modeled with quasi-classical mean-field Mie Monte Carlo $(\hbox {M}^3\hbox {C})$ simulations [10]. Electron trajectories are calculated by integration of classical equations of motion

1 $$\begin{aligned} m \ddot{\mathbf {r}} = -e\underbrace{\left[ \varvec{\mathcal {E}}_\text {Mie}(\mathbf {r},t) - \mathbf {\nabla }\varPhi _\text {mf}(\mathbf {r},t)\right] }_{\varvec{\mathcal {E}}_\text {eff}(\mathbf {r},t)} \end{aligned}$$

in an effective electric field $\varvec{\mathcal {E}}_\text {eff}$, where *e* is the elementary charge and *m* is the electron mass. The effective field consists of the linear response near field $\varvec{\mathcal {E}}_\text {Mie}$ (Mie solution) induced by the infrared few-cycle laser pulse and the field resulting from an instantaneous, self-consistent mean-field potential $\varPhi _\text {mf}$, as described in more detail below. The efficient treatment of the Mie fields is discussed elsewhere [10], including a systematic analysis of the size-dependent relative enhancement and ellipticity. For the generation of trajectories via tunnel ionization, we consider that the tunneling path starts inside the sphere, follows the local electric field $\varvec{\mathcal {E}}_\text {eff}$, and ends at the classical tunneling exit. The average field along the tunneling path is used to calculate the ionization probability via Ammosov-Delone-Krainov atomic tunneling rates [16], assuming an effective ionization potential of $9\,\hbox {eV}$ to describe the band gap of SiO_2_. Successful tunneling events are sampled via Monte Carlo methods, leading to the generation of residual ions and free electrons with zero initial velocity at the start and end points of the respective tunneling paths. The electrostatic mean-field potential $\varPhi _\text {mf}$ is calculated from the instantaneous charge density $\varrho _\text {f}(\mathbf {r},t)$ of free carriers by solving Poisson’s equation

2 $$\begin{aligned} \Delta \varPhi _\text {mf}(\mathbf {r},t) = -\frac{\varrho _\text {f}(\mathbf {r},t)}{\varepsilon _0\varepsilon ^\text {in/out}}, \end{aligned}$$

with the vacuum permittivity $\varepsilon _0$ an the relative permittivities $\varepsilon ^\text {in}=2.12$ and $\varepsilon ^\text {out}=1$ and using multipole expansion. At the sphere surface, the potential $\varPhi _\text {mf}$ must be continuous and has to fulfill the boundary condition $\varepsilon ^\text {in}\frac{\partial }{\partial r} \varPhi _\text {mf}^\text {in} = \varepsilon ^\text {out}\frac{\partial }{\partial r} \varPhi _\text {mf}^\text {out}$. The charge density $\varrho _\text {f}(\mathbf {r}) = \varrho _\text {e}(\mathbf {r}) + \varrho _\text {i}(\mathbf {r})$ is composed of the contributions from activated electrons and residual ions and is evaluated by ensemble averaging over a sufficiently large set of trajectories to achieve convergence. A constant relative permittivity $\varepsilon ^\text {in}$ is used for the calculation of both the Mie fields and the mean-field potential. This approximation neglects that the valence band susceptibility of the dielectric material is reduced due to ionization losses, which is well justified for low average degrees of ionization. The much larger and strongly nonlinear dynamic polarization of the generated free electrons, however, is accounted for in the self-consistent mean-field potential in electrostatic approximation. Finally, elastic and inelastic collisions inside the sphere are evaluated via Monte Carlo sampling using the self-consistent electron trajectories. The elastic electron–atom collisions are treated as instantaneous, isotropic scattering events using an energy-dependent mean-free path that is derived from transport cross sections predicted by quantum mechanical electron–atom scattering calculations. Inelastic electron–electron scattering, i.e., electron impact ionization, is described by Lotz-type impact ionization cross sections [17]. In a successful inelastic scattering event, the energy of the impinging electron is reduced by the effective ionization potential and a new electron ion pair is created. We note that conceptually similar classical trajectory Monte Carlo simulations have also been performed for nanotips [18].

From the perspective of many-particle theory, the self-consistent electron propagation represents a description on the Vlasov level, where the Mie field describes the external driving field. The tunneling ionization as well as the elastic and inelastic collisions represent additional source and collision terms, eventually resulting in a dynamical description equivalent to a Boltzmann equation. Corresponding kinetic transport simulations, including versions that account for limited quantum features, have been applied with great success to the description of intense laser–cluster interactions [19–26].

In the M^3^C model used in the current study, the mean-field term includes the space charge field resulting from ionization and charge separation at the sphere surface and thus accounts for quenching of tunneling ionization, confinement of slow electrons, and Coulomb repulsion in the escaping electron bunches. Further, the inclusion of the sphere polarization in the mean-field potential takes into account image charge effects on the mean-field level.

Emitted electrons are determined from the ensemble of trajectories as those with positive single-particle energy and positive radial velocity. The single-particle energy spectra are evaluated sufficiently late in the simulation to ensure convergence and essentially reflect the electron spectra that would be measured at the detector. To characterize trajectories, we count the number of revisits to the particle, *n*, effectively providing a counter for recollisions. Note that several microscopic collisions may be involved within a single recollision process. For the following analysis, we consider linearly polarized incident pulses with $\tau = 4\,\hbox {fs}$ pulse duration (full width at half maximum of the intensity envelope) at $\lambda = 720\,\hbox {nm}$ central wavelength propagating along the *x*-direction. The incident laser electric field is defined as ${\varvec{\mathcal {E}}}(\mathbf{r},t)={\mathcal {E}}_0\mathbf{e}_y f(t-x/c)\cos (\omega t +\varphi _{\rm CEP}-kx)$, where ${\mathcal {E}}_0$ is the laser peak field strength, *f*(*t*) is a Gaussian temporal envelope, *c* is the vacuum speed of light, $\omega$ is the angular carrier frequency with the corresponding wavenumber *k*, and $\varphi _\text {CE}$ is the CEP. In this work, all laser intensities refer to the incident light field.

## Results and discussion

**Fig. 2:**
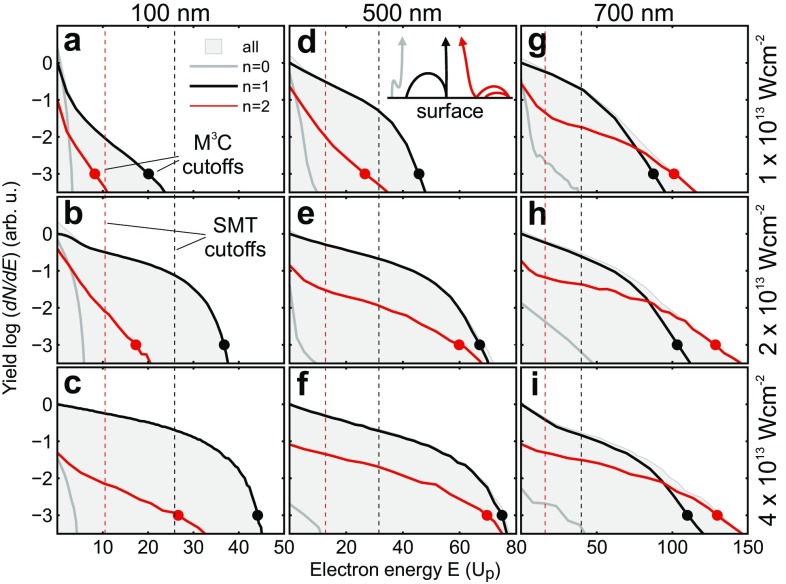
Calculated recollision-resolved energy spectra. Electron energy spectra for three different sphere diameters and laser intensities (as indicated) as predicted by M$^3$C. Spectra for all emitted electrons (*gray shaded areas*) are compared to spectra filtered according to the number of recollision events (*solid curves*) for direct ($n=0$), single ($n=1$), and double ($n=2$) recollision electrons (compare schematic trajectories in **d**). All curves are normalized with respect to the yield of single recollision electrons (*solid black curves*) at ${E} = 0$. *Black* and *red dots* show cutoff energies of single and double recollision electrons, respectively. The cutoffs are defined as the energy where the corresponding normalized yield drops by three orders of magnitude compared to the single recollision yield at ${E} = 0$, i.e., to the level −3 on the logarithmic scale. *Dashed vertical lines* indicate corresponding SMT cutoffs using only the radial component of the Mie field. Note that the energy axis is given in units of the free space ponderomotive potential $U_\text {p}$

The general evolution of the simulated CEP-averaged electron spectra with sphere size and intensity is depicted in Fig. 2. Note that the spectra are plotted as function of the ponderomotive potential $U_\text {p} = e^2{\mathcal {E}}_0^2 / 4 m \omega ^2$ of the incident laser field. Besides the full spectra (gray areas), the selective contributions from different acceleration processes (solid curves) are shown. Direct electrons, i.e., electrons that are born outside via tunnel ionization and do not re-scatter at the particle ($n=0$), appear only in the low-energy region and contribute noticeably to the yield only for low intensities and small particles. The suppression of direct electrons with intensity and size already suggests a substantial trapping of electrons by the ionization induced attractive space charge potential [8]. Due to their low impact on the high-energy region, direct electrons are not of interest for the current study. In contrast, single recollision electrons ($n=1$) constitute the dominant contributions for 100 and 500 nm in the considered intensity range. However, double recollision electrons ($n=2$) become increasingly important with sphere size and prevail the spectra near the cutoff energy for the largest particle size of 700 nm irrespective of intensity. In the following, we investigate the evolution of the yields, cutoff energies, and acceleration dynamics of single and double recollision electrons in detail.

### Evolution of electron yield and cutoff energy

**Fig. 3:**
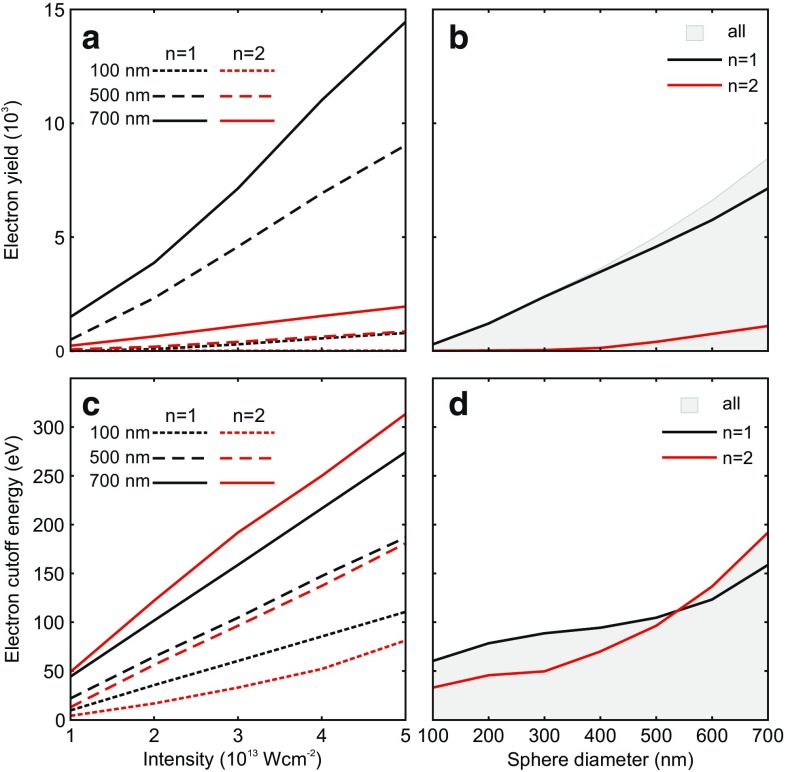
Intensity and size dependence of yields and cutoff energies for electrons emitted from SiO_2_ nanospheres. **a** Single (*black curves*) and double (*red curves*) recollision electron yields as function of laser intensity for three different sphere diameters, as predicted by M^3^C theory. **b** Size-dependent scaling of the total yield (*gray shaded area*) and the yields of single and double recollision electrons for $I=3 \times 10^{13}\,\hbox {Wcm}^{-2}$. The yields include all electrons with positive single-particle energy and positive radial velocity at the end of the simulation and thus reflect the predicted electron yields at the detector. **c** Cutoff energies of single and double recollision electrons as function of the laser intensity for different sphere diameters (as indicated). **d** Evolution of cutoff energies for single and double recollision electrons as function of the sphere size for $I=3 \times 10^{13}\,\hbox {Wcm}^{-2}$

In the framework of the simple man’s theory (SMT) [27], applied to the acceleration of direct and recollision electrons from a surface under the assumption of specular reflection, the total yield of emitted electrons is only determined by the local tunnel ionization rate. As the latter typically increases exponentially with intensity, an exponential growth may also be assumed for the total yield. In stark contrast to this expectation, the yields of both single and double recollision electrons obtained from M^3^C calculations show a nearly linear increase with laser intensity, irrespective of sphere size, as shown in Fig. 3a. This quenching of the emission signifies Coulomb blocking of the tunnel ionization due to charge separation at the particle surface. Further, considering a fixed intensity, the yields scale nearly linearly also with sphere size, as shown in Fig. 3b. This trend can be rationalized with a simplified picture of the space charge trapping effect. Considering the sequential electron emission with fixed initial kinetic energy from the surface of a sphere, the emission is quenched after reaching a total charge $Q_{\rm max}$ for which the emerging attractive Coulomb potential equals the given initial electron energy. Because of the *Q* / *R* dependence of the Coulomb potential, a linear dependence of the number of electrons that can be emitted before the onset of trapping ($Q_{\rm max} \sim R$) is expected, in reasonable agreement with the simulation results. Note that this picture has a direct analogy to the frustrated multistep ionization of clusters in intense X-ray laser fields [28–30]. A quantitative comparison of measured and calculated yields has been performed in Ref. [10] for similar conditions ($d=400\,\hbox {nm}$, $I=3 \times 10^{13}\,\hbox {Wcm}^{-2}$). As the electron yield at low energies (≤10 $\hbox {U}_\text {p}$) may be obscured by contributions from background gas, the comparison has been performed for the high-energy electrons that can be uniquely attributed to the photoemission from the nanospheres. The agreement between experiment and theory supports that the simulations provide a realistic prediction of the total electron yield.

Also the intensity dependent cutoff energies for both single and double recollision electrons exhibit a nearly linear slope for all investigated sphere sizes, as shown in Fig. 3c. While an offset free proportionality ($E_\text {c} \propto I$) is predicted by SMT in the limit of large decay lengths of the near field, the linear fit of the cutoff energy evolution from the full M^3^C simulations shows a notable energy offset that is attributed to the charge interaction effect. However, the most important prediction of the calculations is that for a fixed intensity the cutoff energy of double recollision electrons increases faster with sphere size as for single recollisions, as shown in Fig. 3d. Note that within SMT and when neglecting the tangential field components, the ratio of single to double recollision electron cutoff energy is $\approx$40 % (compare Fig. 2 and [10]). The $\hbox {M}^3$C analysis predicts that for particles $\gtrsim 600\,\hbox {nm}$ the double recollision process becomes clearly dominant near the cutoff, although the total yield of the double recollision contribution remains below that of single recollisions.

### Trajectory analysis of single and double recollisions

**Fig. 4:**
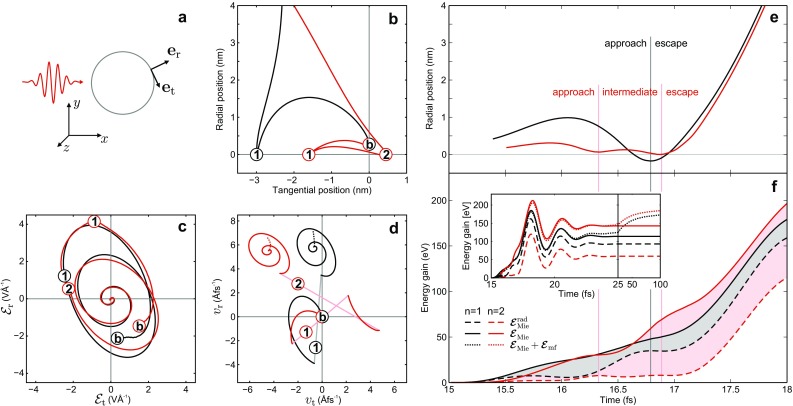
Trajectory analysis of single and double recollision processes. Typical trajectories of single (*black*) and double (*red*) recollision electrons emitted from 700 nm SiO_2_ spheres under a 4-fs pulse at intensity $I=4 \times 10^{13}\,\hbox {Wcm}^{-2}$ centered at 15 fs with $\varphi _\text {CE} = 0$. **a** Definition of unit vectors $\mathbf {e}_\text {r}$ and $\mathbf {e}_\text {t}$, used to calculate radial and tangential contributions of different properties for the trajectories discussed in (**b**–**d**). **b** Evolution of radial versus tangential excursions (with respect to the birth positions) for typical single and double recollision trajectories. The labeled *circles* mark the respective moments of birth (labeled with **b**) and the recollisions (labeled by recollision number) and correspond to (**c**, **d**). Radial offsets at the birth times resemble the classical tunneling exits with respect to the surface. **c** Evolution of the elliptic local near fields. **d** Evolution of radial and tangential velocities. The jumps of the velocities at the recollision events are indicated by *pastel-colored lines*. The *dotted parts* of the curves indicate the additional velocity gain on long time scales ($>$25 fs, compare inset in **f**). **e** Averaged radial trajectories of single and double recollision electrons with final energies near the cutoff ($\pm$1 % of the respective cutoff energy). *Vertical lines* indicate the average times of the recollision events. **f** Selective energy gains $\Delta E(t) = \int _{t_\text {b}}^{t} \dot{\mathbf{r}}(t')\cdot \varvec{\mathcal {E}}[\mathbf{r}(t')]dt'$ from different field contributions $\varvec{\mathcal {E}}$ for the trajectories in **e** during the recollision process (see *inset* for full time evolution). *Gray* and *red areas* indicate the energy gains from only the tangential field for single and double recollision electrons, respectively

In order to unravel the origin of the increasing importance of the double recollision process in the cutoff region for large nanospheres, we performed a systematic trajectory analysis for the largest investigated sphere size of $d=700\,$nm. Therefore, we traced typical trajectories of single and double recollision electrons with final energies close to the respective cutoffs and analyzed the evolution of position, local electric field, and velocity (Fig. 4b–d). To analyze the radial and tangential components of the latter three properties, we project on radial and tangential unit vectors with orientations indicated in Fig. 4a. The single recollision trajectory resembles the well-known physics of the single recollision process. After birth at the classical tunneling exit (circle labeled with b in Fig. 4b), it exhibits a large radial excursion and an almost purely radial recollision near the zero crossing of the radial field (compare black circles labeled with 1 in Fig. 4b, c). This allows for efficient radial acceleration during the approach and the escape phase, accompanied by an almost purely radial jump of the velocity at the moment of recollision (black circle labeled with 1 in Fig. 4d).

The double recollision trajectory reveals a substantially different acceleration mechanism. Compared to the single recollision trajectory, the radial excursion is much smaller during the whole recollision process. Instead, it exhibits a pronounced tangential motion in between the two recollisions (red circles labeled with 1 and 2 in Fig. 4b). Note, that the second recollision is timed similarly as the recollision in the single recollision process, i.e., near the zero crossing of the radial field, allowing for an efficient radial acceleration during the escape phase. However, the first recollision near the zero crossing of the tangential field (red circle labeled with 1 in Fig. 4c) turns out to be the pivotal point for the efficiency of the double recollision process. Substantial tangential acceleration is possible if the velocity vector remains oriented antiparallel to the tangential electric field, i.e., if the tangential velocity vector is flipped at the first recollision event and if the tangential field flips as well. Note that this scenario is realized in the displayed typical trajectory, as shown in red circles labeled with 1 and 2 in Fig. 4d.

In the next step, we inspect the temporal evolution of averaged trajectories in relation to the energy absorption. The evolution of the averaged single recollision trajectory in Fig. 4e shows the typical backscattering scenario with a small but finite ($<$1 nm) penetration of the sphere during the reflection process. Note that the smooth evolution results from the trajectory averaging while the scattering for the underlying individual trajectories proceeds abruptly. The averaged double recollision trajectory shows two reflection processes delayed by about half a femtosecond, with the timing of the final reflection similar (difference $\approx 150\,\hbox {as}$) to the single recollision process.

The above picture is corroborated by an analysis of the single-particle energy gains from different field contributions, as shown in Fig. 4f. This kind of analysis already revealed the impacts of mean-field and field ellipticity on the almost purely radial acceleration process of single recollision electrons [10]. The latter can efficiently gain energy from the radial component of the Mie field during the approach and the escape phase of the recollision process (separated by the vertical black line in Fig. 4e, f) as illustrated by the respective steeper slopes of the dashed black curve in Fig. 4f. In comparison, the additional energy gain from the tangential Mie field (gray area) is relatively small.

In contrast, for the double recollision process, the analysis reveals that the energy gain from the radial Mie field (dashed red) is small during the approach phase and between the first and second recollision (phases separated by vertical red lines). Only during the escape phase, i.e., after the second recollision that is similarly timed to the recollision in the single recollision process, the energy gain from the radial Mie field is high. However, the energy gain from tangential components of the Mie field (red area, i.e., the difference between solid and dashed red curves) increases during the approach and between the two recollisions. This additional gain is the reason for the dominance of the double recollision process in the energy cutoff region for large spheres. Note that the gain for the full near field does not change much when compared to the Mie field induced energy gain during the recollision process (compare solid and dotted curves for small times in the inset of Fig. 4f). This signature can be explained as follows. The short-range effect of the mean field is the emergence of an approximately static trapping potential that affects the trajectories but does not contribute to the energy gain directly. Hence, when comparing to SMT results (not shown), the trapping potential induced higher energy gain on the short time scales results just from the modified trajectory in the Mie field. However, on a longer timescale, the energy gain associated with the mean field does contribute notable to both single and double recollision electrons due to Coulomb explosion of the escaping bunches [10]. Note that the corresponding changes of the velocities due to the latter effect are indicated by the dotted ends of the curves in Fig. 4d and are almost purely radial for the selected typical trajectories.

### Emission directionality of recollision electrons

**Fig. 5:**
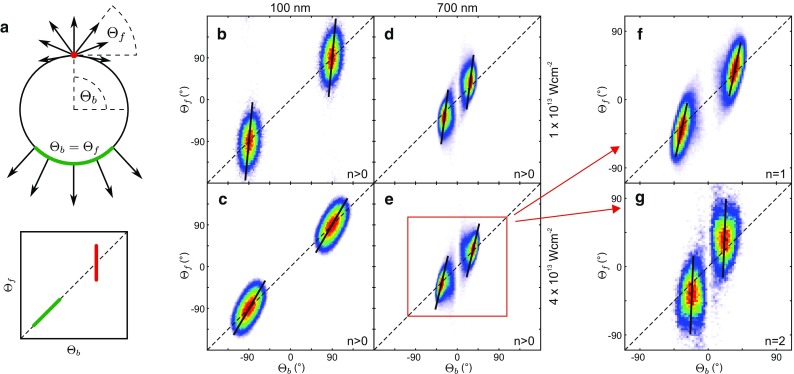
Correlation analysis of the electron emission. **a** Schematic representation of correlation characteristics between birth angle $\varTheta _\text {b}$ and final angle $\varTheta _\text {f}$, defined via the projections of birth position and final momentum vector on the *x*–*y* and $p_x-p_y$ planes, respectively, for undirected (*red*) and radial (*green*) emission. **b**–**e** Correlation plots for fast ($E>0.5E_\text {c}$) recollision electrons ($n>0$) emitted from small and large spheres at low and high intensities (as indicated). The *dashed black diagonals* represent the case of radial emission where $\varTheta _\text {b} = \varTheta _\text {f}$. *Solid black lines* are guides to the eye to indicate the tilts of the distributions. **f, g** Enlarged representation of the indicated area in **e** for single and double recollision electrons, respectively

So far we identified the impact of the tangential field on the photoelectron acceleration dynamics and energy spectra. In the next step, we study the tangential field effect on the emission directionality and on the CEP dependence of the electron emission. Therefore, we analyze the correlation of birth angle $\varTheta _b$ and final emission angle $\varTheta _f$ for each electron trajectory (Fig. 5a). Two important limiting cases of this correlation analysis can be distinguished. Undirected photoemission from a narrow birth angle region but with a broad distribution of final angles will appear as a vertical feature in the correlation plot (marked in red in Fig. 5a). In contrast, a concentration of trajectories on the diagonal (marked in green in Fig. 5a) signifies radial emission that allows to directly map emission to birth angles.

For small nanospheres, where tangential field components are negligible, the correlation analysis shows a transition from undirected to radial emission as intensity increases (Fig. 5b, c). This signature is attributed to the trapping potential near the surface induced by the mean field, which acts as a filter that enhances radial emission. Only electrons with sufficiently high radial velocities can overcome the attractive trapping potential near the surface. Although this behavior is still visible for large spheres, it is much less pronounced, as shown in Fig. 5d, e. This trend indicates the suppression of trajectories with effectively purely radial motion because of the presence of significant alternating tangential velocity components for all trajectories, underlining the growing importance of tangential acceleration effects.

To disentangle the impact of these effects on the directionality for the single and double recollision processes separately, Fig. 5f, g shows corresponding filtered correlation plots. The distribution for the double recollision process has a nearly vertical shape and exhibits a stronger offset from the diagonal, i.e., is shifted toward higher emission angles when compared to the single backscattering analysis. Both features document the stronger tangential acceleration and a broader angular spread in the double recollision process. In conclusion, the directional filtering effect of the mean field is most pronounced for single recollision electrons and small spheres. The suppression of this effect with large spheres, especially for double recollision electrons, can be explained with the growing impact of the tangential fields.

**Fig. 6:**
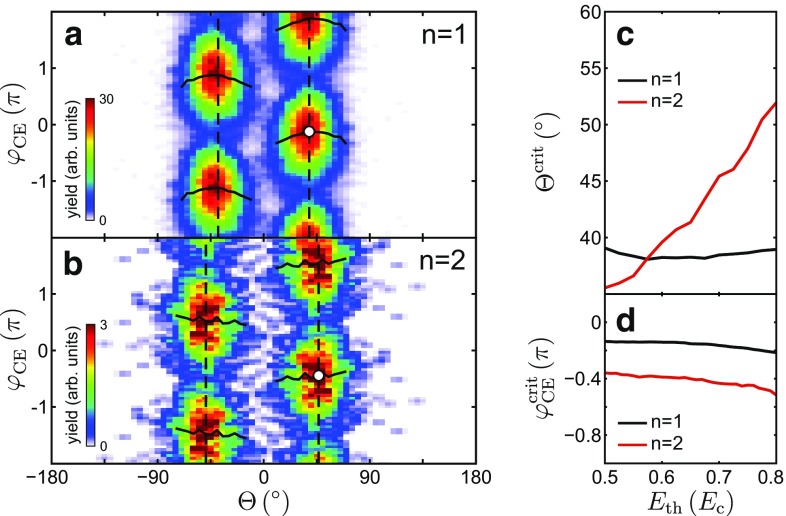
Phase-dependent switching and emission directionality. **a, b** Directionality and phase dependence of the calculated yields of fast ($E>E_\text {th} = 0.75 E_\text {c}$) single (**a**) and double (**b**) recollision electrons emitted from $700\,\hbox {nm}$ SiO_2_ at $4\times 10^{13}\,\hbox {Wcm}^{-2}$. *Vertical dashed lines* illustrate critical emission angles $\varTheta ^\text {crit}$ for upward and downward emission; *horizontal solid lines* show fitted phase offsets $\Delta \varphi (\varTheta )$ and *white dots* the critical CEPs $\varphi _\text {CE}^\text {crit}$ (as introduced in the caption of Fig. 1d–g). **c, d** Evolution of the critical angles and CEPs from (**a**, **b**) as function of the respective threshold energy for single and double recollision electrons

Finally, we study the laser waveform effects on the emission of single and double recollision electrons for a large particle. The CEP dependence of the angular-resolved yields for fast electrons (Fig. 6a, b) shows a qualitatively similar behavior for both processes. This supports that CEP-controlled switching remains feasible also for the double recollision process. However, the critical phases and critical angles for single and double recollision electrons (determined as in Fig. 1d–g) show a small but notable deviation. To also test the energy dependence of the critical phases and critical angles, we further varied the threshold energy, as shown in Fig. 6c, d. The critical angle of single recollision electrons in Fig. 6c is almost independent of the threshold energy, underlining the dominance of the radial recollision dynamics for this class of trajectories [10]. In contrast, the critical angle for double recollision electrons shifts more than 15 degrees toward the particle poles when varying the threshold. This confirms the much stronger sensitivity of double recollisions on the tangential field. For both single and double recollision electrons, the critical CEPs (Fig. 6d) are almost independent of the threshold energy. In fact, the critical phase even slightly decreases with increasing threshold energy while the opposite trend was found for small particles [9]. Note that the nearly constant offset between the critical phases and similar CEP-dependent signal modulation indicate an effectively similar switching behavior of the two recollision processes.

## Conclusions

We investigated the strong-field induced photoemisson from dielectric nanospheres as function of intensity and size. Our study shows that the yields of single and double recollision electrons exhibit nearly linear dependencies with both intensity and size, which can be explained as a result of space charge trapping in the nanosphere’s Coulomb potential. The analysis further shows that double recollision electrons become important for large spheres because of the increasing relative strength of tangential field components. Our trajectory analysis provides a simple picture to explain the enhancement of the double recollision acceleration process. The single and double recollisions are timed to the zero crossings of the tangential and radial field, respectively, and therefore allow efficient energy gain from the corresponding field components. Our analysis further supports that the single recollision process remains dominated by the radial field evolution while the double recollision process is highly sensitive to the tangential field. Although the CEP-dependent emission directionality is qualitatively similar for the single and double recollision processes, in the energy region beyond the single recollision cutoff the signatures from double recollision electrons tilt toward the particle poles with increasing energy. Therefore, based on our results, the double recollision process may be identified experimentally in the energy spectra or via a threshold energy-dependent analysis of the emission directionality. In conclusion, we identified a so far unresolved interplay of charge interaction and field ellipticity in the near-field induced photoemission and we expect that our findings are relevant for the strong-field induced electron emission from any (nano-)target with nontrivial near-field structure and evolution. The processes should therefore also be relevant for nanotips, for which the so far published results mainly considered locally linearly polarized near fields and the regime of near single electron emission per pulse. The utilization of multielectron effects and elliptic near fields for controlling recollision phenomena in laser–nanotarget interactions is thus an interesting direction for future studies.

## References

[1] Gramotnev D, Bozhevolnyi S (2014). Nat. Photon..

[2] Krausz F, Stockman MI (2014). Nat. Photon..

[3] Krüger M, Schenk M, Hommelhoff P (2011). Nature.

[4] Herink G, Solli DR, Gulde M, Ropers C (2012). Nature.

[5] Piglosiewicz B, Schmidt S, Park DJ, Vogelsang J, Groß P, Manzoni C, Farinello P, Cerullo G, Lienau C (2014). Nat. Photon..

[6] Park DJ, Piglosiewicz B, Schmidt S, Kollmann H, Mascheck M, Groß P, Lienau C (2013). Ann. Phys..

[7] Krüger M, Schenk M, Hommelhoff P, Wachter G, Lemell C, Burgdörfer J (2012). New J. Phys..

[8] Zherebtsov S, Fennel T, Plenge J, Antonsson E, Znakovskaya I, Wirth A, Herrwerth O, Süßmann F, Peltz C, Ahmad I, Trushin SA, Pervak V, Karsch S, Vrakking MJJ, Langer B, Graf C, Stockman MI, Krausz F, Rühl E, Kling MF (2011). Nat. Phys..

[9] Zherebtsov S, Süßmann F, Peltz C, Plenge J, Betsch KJ, Znakovskaya I, Alnaser AS, Johnson NG, Kübel M, Horn A, Mondes V, Graf C, Trushin SA, Azzeer A, Vrakking MJJ, Paulus GG, Krausz F, Rühl E, Fennel T, Kling MF (2012). New J. Phys..

[10] Süßmann F, Seiffert L, Zherebtsov S, Mondes V, Stierle J, Arbeiter M, Plenge J, Rupp P, Peltz C, Kessel A, Trushin SA, Ahn B, Kim D, Graf C, Rühl E, Kling MF, Fennel T (2015). Nat. Commun..

[11] Dombi P, Hörl A, Rácz P, Márton I, Trügler A, Krenn JR, Hohenester U (2013). Nano Lett..

[12] Paulus GG, Nicklich W, Xu H, Lambropoulos P, Walther H (1994). Phys. Rev. Lett..

[13] Wimmer L, Herink G, Solli DR, Yalunin SV, Echternkamp KE, Ropers C (2014). Nat. Phys..

[14] Feist A, Echternkamp KE, Schauss J, Yalunin SV, Schäfer S, Ropers C (2015). Nat.

[15] Radcliffe P, Arbeiter M, Li WB, Düsterer S, Redlin H, Hayden P, Hough P, Richardson V, Costello JT, Fennel T, Meyer M (2012). New J. Phys..

[16] Ammosov MV, Delone NB, Krainov VP (1986). Sov. Phys. JETP.

[17] Lotz W (1967). Z. Phys..

[18] Wachter G, Lemell C, Burgdörfer J, Schenk M, Krüger M, Hommelhoff P (2012). Phys. Rev. B.

[19] Fennel T, Döppner T, Passig J, Schaal C, Tiggesbäumker J, Meiwes-Broer K-H (2007). Phys. Rev. Lett..

[20] Fennel T, Bertsch G, Meiwes-Broer K-H (2004). Eur. Phys. J. D.

[21] Fennel T, Meiwes-Broer K-H, Tiggesbäumker J, Reinhard P-G, Dinh PM, Suraud E (2010). Rev. Mod. Phys..

[22] Köhn J, Redmer R, Meiwes-Broer K-H, Fennel T (2008). Phys. Rev. A.

[23] Köhn J, Fennel T (2011). Phys. Chem. Chem. Phys..

[24] Köhn J, Redmer R, Fennel T (2012). New J. Phys..

[25] Calvayrac F, Reinhard P-G, Suraud E, Ullrich C (2000). Phys. Rep..

[26] Domps A, Reinhard P-G, Suraud E (1998). Phys. Rev. Lett..

[27] Corkum PB (1993). Phys. Rev. Lett..

[28] Bostedt C, Thomas H, Hoener M, Eremina E, Fennel T, Meiwes-Broer K-H, Wabnitz H, Kuhlmann M, Plönjes E, Tiedtke K, Treusch R, Feldhaus J, de Castro ARB, Möller T (2008). Phys. Rev. Lett..

[29] Arbeiter M, Fennel T (2010). Phys. Rev. A.

[30] Schütte B, Campi F, Arbeiter M, Fennel T, Vrakking MJJ, Rouzée A (2014). Phys. Rev. Lett..

